# Meeting at Plymouth

**Published:** 1918-03-30

**Authors:** 


### THE NATION'S FUND FOR NURSES.

### Meeting at Plymouth.

A meeting, at which Sir Arthur Stanley, Chairman of the Joint War Committee of the British Red Cross Society and the Order of St. John of Jerusalem, was the principal speaker, was held at Plymouth on March 21 in aid of the "Nation's Fund for Nurses," under the presidency of the Mayor. The meeting was organised by Miss McKay, R.R.C., matron of the 4th Southern General Hospital, Salisbury Road, and was attended by a large company of matrons, sisters, and nurses and a representative gathering.

The Mayor, who was accompanied by Mrs. Brown, spoke of the indebtedness of the nation to the nurses not only in this time of war, but at all times. This movement enabled them to show their appreciation in a tangible way. He was convinced the movement was on thoroughly sound lines.

Sir A. Stanley said the promoters of the College of Nursing had no axe to grind and no object in view except the welfare of the nursing profession, and they were going to stick steadily to that object, whatever anybody might say or think. If they did that he was convinced of success; in fact, they had got so far that it might well be said they had succeeded already in providing a strong, definite, powerful, central organisation for the nursing profession. They were prepared to meet any other nursing society on the subject of the State Registration Bill. The Council would be elected by the members of the College themselves by postal ballot. With the College there would also be a benevolent fund for the nursing profession. The National Pension Fund for Nurses was a purely business institution. He could not find there was any other skilled profession in the world which had no benevolent fund. This was to be called "The Tribute Fund," because it was to be the tribute of the people of the British Empire to the nurses who had worked so gloriously in this war. (Applause.) Complaint was made that it would be "charity"; but did officers and men who had been under the care of the Red Cross call it "charity"? The endowment of the College of Nursing and the establishment of this Tribute Fund were included in what was called "The Nation's Fund for Nurses." There was an impression abroad, fostered, he feared, by their enemies, that this fund on its benevolent side was meant to help those nurses only who were members of the College. That was absolutely untrue. If there was any woman who had the right to call herself a nurse, whether she belonged to the College or not, if she was in need or in sickness she would only have to come to that fund and they would do their best to help her. (Applause.) It was intended that the College should not only have a great centre in London; they wanted a great building in London to be a real centre for the nursing profession not only of these islands, but for the British Dominions beyond the seas. That would be of no use unless surrounded by centres in all principal towns such as Plymouth. (Hear, hear.) This was the moment they must not miss. We had a vast army ashore and afloat fighting for our liberties, facing death, disease, and suffering; and there was a much smaller army, equally devoted and loyal—that was the army of nurses—who were fighting against suffering and disease and doing everything they could to relieve the appalling nature of this war. (Applause.) Was it not up to us to do what we could to help them in their work? Having seen more than any other man of the work of the nurses in this war, he should feel ashamed if, when this war was over, we could not offer the nurses a real live organisation capable of protecting their interests, and of speaking in their name, in order that all that could be done for the nurses should be done. (Loud applause.)

Mrs. Waldorf Astor said that the salaries of nurses in institutions were a scandal. She believed she had seen more of nurses even than Sir A. Stanley. Scarcely a day had passed since the war began that she had not been in some hospital. The patience of nurses, speaking generally, was wonderful, and they deserved everything we could do for them. She concluded by proposing thanks to "that noble and long-suffering person," the Mayor.

